# Quercetin inhibits SARS-CoV-2 infection and prevents syncytium formation by cells co-expressing the viral spike protein and human ACE2

**DOI:** 10.1186/s12985-024-02299-w

**Published:** 2024-01-25

**Authors:** Annie V. Roy, Michael Chan, Logan Banadyga, Shihua He, Wenjun Zhu, Michel Chrétien, Majambu Mbikay

**Affiliations:** 1 Functional Endoproteolysis Laboratory, Montreal Clinical Research Institute, Montreal, QC Canada; 2 Special Pathogens Program, National Microbiology Laboratory, Public Health Agency of Canada, Winnipeg, MB Canada; 3 Department of Medical Microbiology and Infectious Diseases, University of Manitoba, Winnipeg, MB Canada; 4 National Centre for Foreign Animal Disease, Canadian Food Inspection Agency, Winnipeg, MB Canada

**Keywords:** Quercetin, SARS-CoV-2, Spike protein, Human ACE2, HEK293 cells, Syncytium formation

## Abstract

**Background:**

Several *in silico* studies have determined that quercetin, a plant flavonol, could bind with strong affinity and low free energy to SARS-CoV-2 proteins involved in viral entry and replication, suggesting it could block infection of human cells by the virus. In the present study, we examined the ex vivo ability of quercetin to inhibit of SARS-CoV-2 replication and explored the mechanisms of this inhibition.

**Methods:**

Green monkey kidney Vero E6 cells and in human colon carcinoma Caco-2 cells were infected with SARS-CoV-2 and incubated in presence of quercetin; the amount of replicated viral RNA was measured in spent media by RT-qPCR. Since the formation of syncytia is a mechanism of SARS-CoV-2 propagation, a syncytialization model was set up using human embryonic kidney HEK293 co-expressing SARS-CoV-2 Spike (S) protein and human angiotensin converting enzyme 2 (ACE2), [HEK293(S + ACE2) cells], to assess the effect of quercetin on this cytopathic event by microscopic imaging and protein immunoblotting.

**Results:**

Quercetin inhibited SARS-CoV-2 replication in Vero E6 cells and Caco-2 cells in a concentration-dependent manner with a half inhibitory concentration (IC_50_) of 166.6 and 145.2 µM, respectively. It also inhibited syncytialization of HEK293(S + ACE2) cells with an IC_50_ of 156.7 µM. Spike and ACE2 co-expression was associated with decreased expression, increased proteolytic processing of the S protein, and diminished production of the fusogenic S2’ fragment of S. Furin, a proposed protease for this processing, was inhibited by quercetin in vitro with an IC_50_ of 116 µM.

**Conclusion:**

These findings suggest that at low 3-digit micromolar concentrations of quercetin could impair SARS-CoV-2 infection of human cells partly by blocking the fusion process that promotes its propagation.

**Supplementary Information:**

The online version contains supplementary material available at 10.1186/s12985-024-02299-w.

## Background

Severe Acute Respiratory Coronavirus 2 (SARS-CoV-2) is the etiological agent of coronavirus disease 2019 (COVID-19) that broke out in 2019 in China before becoming a pandemic that has killed to date nearly 15 million people worldwide [1]. SARS-CoV-2 primarily infects epithelial cells of respiratory airways where it multiplies, releasing virions that disseminate to other organs including heart, liver, bowel, kidney, and brain. Infection by the virus often elicits from the host an inflammatory response that can evolve into a potentially fatal “cytokine storm”. The most common symptoms of COVID-19 include fever, cough, fatigue, anosmia and ageusia; common symptoms include pharyngitis, cephalgia, myalgia, iritis, or diarrhea; complications include dyspnea, pneumonia, severe acute respiratory distress syndrome, hypercoagulation syndrome, and multiple organ failure [2]. The global case fatality rate of COVID-19 was estimated at 8.5% in February 2020; it had decreased to 0.27% by August 2022 [3]. It comprises, for a large part, the elderly and individuals with preexisting morbidities such as chronic obstructive pulmonary disease, coronary heart disease, diabetes mellitus, and hypertension [4, 5].

SARS-CoV-2 entry into cells begins with the binding of the viral Spike (S) protein to human angiotensin converting enzyme 2 (ACE2), followed by the proteolytic cleavage of S by furin into the S1 ectodomain and the S2 transmembrane domain. Further cleavage of the S2 fragment by either furin or transmembrane protease serine 2 (TMPRSS2) exposes the fusion peptide at the amino terminus of the transmembrane S2’ domain, initiating the fusion of the viral envelope to the cell plasma membrane and the entry of the virus unto the cell. In cellular lysosomes, the virus is unpacked into its RNA and protein components, a process facilitated by cathepsins B and L (CatB and CatL); the RNA is translated into a polyprotein which is cleaved by viral main protease (M^pro^), papain-like protease (PL^pro^), and 3 chymotrypsin-like protease (3CL^pro^) to generate functional proteins; the RNA is replicated by viral RNA-dependent RNA polymerase (RdRp); the new RNA is translated to produce new proteins; these RNA and proteins are assembled into virions which are released in the extracellular milieu [2].

Proteins that facilitate viral entry and replication are potential targets for inhibition of SARS-CoV-2 infection. Viral entry can be interfered with by blocking the binding of S protein to ACE2 using either soluble decoy ACE2 or anti-S antibodies. RNA or protein vaccines eliciting endogenous anti-S neutralizing antibodies have been approved by regulatory agencies after conclusive clinical trials. Their use in mass immunization of populations around the world has gone a long way in controlling the COVID-19 pandemic by reducing its severity and lethality [6]. Besides these prophylactic measures, potential therapeutic drugs targeting various enzymes in the viral life cycle have been investigated, among them inhibitors of TMPRSS2 (e.g. camestat) [7, 8], cathepsins (e.g. hydroxychloroquine) [9, 10], 3CL^pro^ (e.g. ritanovir, nirmatrelvir) [11], and RdRp (e.g. remdesivir) [12, 13]. Paxlovid, a combination of ritanovir and nirmatrelvir, is now an approved oral drug against early stages of COVID-19 [14].

Interestingly, quercetin, a plant flavonol, could also be an anti-COVID-19 drug. Indeed, molecular docking analyses have indicated that it can bind with relatively strong affinity to several SARS-CoV-2 proteins including S protein, 3CL^pro^, and RdRp [15], as well as to cellular ACE2 [16] and TMPRSS2 [17]. Moreover, it has been shown to inhibit to varying degrees the in vitro enzymatic activities of furin [18], ACE2 [19], 3CL^pro^ [20], M^pro^ [21], and RdRp [22]. Besides these possible interferences with the infection by SARS-CoV-2, quercetin is also likely to attenuate COVID-19 through its recognized potent antioxidant and anti-inflammatory properties [23], since oxidative stress and inflammation are prominent features of the pathophysiology of the disease [24].

Despite these promising indications, very few studies have experimentally explored the activities of quercetin against SARS-CoV-2 infection. Here we report that, at low 3-digit micromolar concentrations, this flavonol reduced SARS-CoV-2 replication in Vero E6 and Caco-2 cells. Furthermore, using syncytium formation by HEK293 cells co-expressing the viral S protein and human ACE2 as a surrogate model for the cytopathic effect that follows SARS-CoV-2 infection, we show that, at viral replication-inhibiting concentrations, quercetin prevented syncytium formation, presumably by decreasing the proteolytic conversion of the S protein to the fusogenic S2’ fragment.

## Methods

### Reagents

Uncommon reagents, their commercial sources and catalog numbers are presented in Supplementary Table S1.

### Cell culture

African green monkey kidney Vero E6 cells and human embryonic kidney 293 (HEK293) cells were cultured in Dulbecco’s Modified Eagle Medium **(**DMEM) supplemented with 10% heat-inactivated fetal bovine serum (hiFBS), 1% L-Glutamine, and 1% penicillin/streptomycin (PeniStrept). Human colon carcinoma Caco2 cells were cultured in Minimal Essential Medium (MEM) containing the same supplements as described for Vero E6 cells. These cell culture media are hereafter referred to as complete media. All cells were cultured at 37 °C in a 5% CO2 atmosphere.

### SARS-CoV-2 infections of Vero E6 and Caco-2 cells

Quercetin was dissolved in DMSO and then diluted to various concentrations in treatment medium (DMEM or MEM, plus 2% hiFBS, 1% L-Glutamine, 1% PeniStrept). VeroE6 and Caco2 cells were cultured in triplicate in 24-well plates in complete medium. When the cells reached 80–90% confluency, medium was removed and replaced with 500 µl treatment medium. Following a 1 h incubation, cells were infected by addition of 250 µl of medium containing SARS-CoV-2/Canada/ON-VIDO492 01/2020 (GISAID accession # EPI_ISL_425177) for a multiplicity of infection (MOI) of 0.1. The final concentrations of quercetin in each replicate were 6.25, 12.5, 25, 50, 100, 200, 400, and 800 µM, and the final concentration of DMSO was 0.3% v/v. Control cells were treated with an equivalent volume of treatment medium containing 0.3% v/v DMSO but no quercetin. Cells were incubated at 37 °C in a 5% CO_2_ for 48 h before supernatant was harvested for the quantification of viral RNA.

For quantification of infectious SARS-CoV-2, Vero E6 cells were seeded in 96-well plate such that confluency was ~ 95% the following day; supernatants from two independent drug treatment studies (performed on both Vero E6 and Caco2 cells) were serially diluted 10-fold in DMEM containing 2% hiFBS and 1% L-glutamine, and 100 µl of each dilution was used to inoculate cells in triplicate; cells were incubated at 37 °C and 5% CO_2_ for seven days, after which they were assessed for cytopathic effect (CPE). The median tissue culture infectious dose (TCID_50_) per ml was calculated for each supernatant sample using the Reed and Muench method. Because the highest concentration (800 µM) of quercetin used in Caco2 cells resulted in CPE in one of three wells at the lowest dilution used (10 − 1), we were unable to calculate the TCID50 for this sample. Instead, the sample was assigned the value of the limit of detection, which was calculated by assuming that a 100 dilution would have produced three of three wells with CPE.

All work with infectious virus was performed in the containment level 3 (CL-3) facility at the National Microbiology Laboratory (NML) of the Public Health Agency of Canada (PHAC) in the Canadian Science Centre for Human and Animal Health (CSCHAH), Winnipeg, Canada. All procedures were conducted in accordance with standard operating protocols appropriate for this level of biosafety.

### Transfection of HEK293 cells

Expression vectors include the following plasmids: (**i**) pEGFP-C1 for expression eGFP (hereafter abbreviated as pGFP); (**ii**) pcDNA3.1-hACE2-C9 for expression of human ACE2 with a C9 tag at its C-terminus (hereafter abbreviated as pACE2); (**iii**) 2019-nCov-Linker_pcDNA3.1(+)-C-eGFP for expression of the SARS-CoV-2 spike protein fused though a linker peptide to a C-terminal enhanced green fluorescent protein (eGFP) (hereafter abbreviated as pS-GFP); (**iv**) 2019-nCov_pcDNA3.1(+)-P2A-eGFP for separate expression the Spike protein and the eGFP (hereafter abbreviated as pS-P2A-GFP). A P2A peptide placed immediately upstream of a glycine causes the ribosome to skip the formation of a peptide bond between the glycine and the next amino acid [25], allowing Spike and eGFP to be translated at the same time but as two unfused proteins. Most experiments were conducted with two expression vectors. When a single expression vector was used, pcDNA-3 was supplemented as an empty vector (EV) to equalize the amount of transfected DNA.

Plasmid transfection was conducted using jetPRIME® Transfection Reagent per manufacturer’s protocol. Transfection vectors were either a 1:1 mix of pACE2 and pS-GFP, a 1:1 mix of pACE2 and pGFP, or 1:1 mix of pS-GFP and pcDNA3 as EV. In a typical experiment, cells were cultured in complete medium as monolayers in 96-well plates; spent media was removed and replaced with 100 µl/well of a 1:9 mixture of jetPRIME® and complete medium. After a 4-h incubation, the transfection medium was removed; cell monolayers were rinsed with complete medium; they were then supplemented with complete medium containing 0.2% DMSO (control) or varying micromolar concentrations of quercetin and 0.2% DMSO; they were further incubated for 16 h.

### IncuCyte capture of syncytium formation and fluorescence

Cells were seeded onto 96-well black/clear bottom plates (Corning) at a density of 30,000 cells/well in triplicates for each condition. After a 24-h incubation, they were transfected, processed, and treated as described above under “Transfection of HEK293 cells”. The IncuCyte® S3 Live-Cell Analysis System (Essen BioScience) was used to capture images of eGFP-emitted fluorescence from each well every 4 h for up to 48 h with a 4× objective, a Green/Red 4614 optic module, and 300 ms exposure. Syncytialization inhibition was accompanied by a decrease in fluorescence. To determine the half inhibitory concentration (IC_50_) of quercetin on syncytium formation, integrated intensity of fluorescence [Green Calibrated Unit (GCU) x µm²/well) at 20 h post-transfection (16 h post-treatment) was quantified and normalized against that of DMSO-treated control cells.

### Confocal microscopy

Transfected HEK293 cells were seeded in an 8-chamber slide at a density of 82 500 cells/chamber and incubated for 24 h. They were fixed with 4% paraformaldehyde for 20 min, washed twice with phosphate buffered saline (PBS), incubated with Hoechst (diluted 1:7500 in PBS) for 10 min to stain DNA, washed again twice, and then overlaid with 300 µl of PBS/chamber. Images were acquired using the AxioObserver LSM700 inverted confocal microscope; (Zeiss) with a 20× objective and processed using the Zen 3.4 software. Excitation/emission wavelengths were 405/435 nm for Hoechst and 488/518 nm for eGFP. For best localize eGFP fluorescence in syncytia, the laser power at 488 nm had to be doubled for HEK293(S + ACE2) cells treated with 0 to 100 µM quercetin to compensate for the reduced fluorescence due to cell fusion. Images were captured with a resolution of 2048 × 2048 pixels.

### Cell viability assay

Quercetin cytotoxicity was assessed in VeroE6 and Caco2 cells. Quercetin was dissolved in DMSO and then diluted to various concentrations in treatment medium (DMEM or MEM, plus 2% hiFBS, 1% L-Glutamine, 1% PeniStrept). Cells were seeded in triplicate in a 96-well plate. Upon reaching 80–90% confluency, medium was removed and replaced with 100 µl treatment medium. Following a 1-h incubation at 37 °C, an additional 50 µl of treatment medium was added. The final concentrations of quercetin were 6.25, 12.5, 25, 50, 100, 200, and 400 µM, and the final concentration of DMSO in each replicate was 0.3% v/v. Control cells were treated with an equivalent volume of treatment medium containing 0.3% v/v DMSO but no quercetin. After 48 h of drug treatment, cells were washed once with PBS, and cell viability was assessed using the CyQUANT™ XTT Cell Viability Assay kit according to manufacturer’s directions.

HEK293 cells were seeded in a 96-well plate at a density of 3 × 10^4^ cells/well and incubated for 24 h; the medium was supplemented with DMSO to a final concentration of 0.2% (v/v, controls) or to varying micromolar concentrations of quercetin and final 0.2% DMSO; incubation was resumed for 24 h. Cell viability was measured using the Abcam MTS Cell Proliferation Assay kit. The assay is based on the reduction of a tetrazolium salt to a colored formazan by live cells. Briefly, complete media containing DMSO or quercetin and DMSO was removed and replaced with 110 µl/well of a 1:9 mix of MTS reagent: complete medium; the plate was incubated at 37 °C for 180 min; the plate was shaken at 600 rpm for 30 s at room temperature (23 °C) and the optical density (OD) at 490 nm was measured for each well using a Multiskan Spectrum plate reader (Thermo Electron Corporation). The average OD of wells without cells was subtracted from the ODs of cell-containing wells. All assays were conducted in triplicates.

### Quantification of SARS-CoV-2 RNA

SARS-CoV-2 RNA was extracted from cell culture supernatant using the QIAamp Viral RNA Mini Kit according to the manufacturer’s instructions. Viral RNA was quantified by reverse transcription quantitative PCR (RT-qPCR) using the LightCycler 480 RNA Master Hydrolysis Probes kit and the QuantStudio3 thermal cycler. Primer and probe sequences were specific for the SARS-CoV-2 *E* gene:

forward primer, 5’-ACAGGTACGTTAATAGTTAATAGCGT-3’;

reverse primer, 5’-ATATTGCAGCAGTACGCACACA-3’; and.

probe, 5’-FAM-ACACTAGCCATCCTTACT GCGCTTCG-BBQ-3’.

Cycling conditions were as follows: 63 °C for 3 min and 95 °C for 30 s, followed by 45 cycles of 95 °C for 15 s and 60 °C for 30 s.

### In vitro assay for spike-ACE2 interaction

On a 96-well plate, each well was coated with 50 µl of 1 µg/ml of Spike Protein overnight at 4 °C; wells were then washed and blocked. Stock solutions of test compounds were prepared: quercetin at 10 mM in DMSO and anti-Spike antibody at 500 nM in PBS. To each well, 10 µl of diluted test compound and 20 µl of 1× immune buffer (20 mM Tris, 50 mM NaCl, 0.5% NP40, 0.5% sodium deoxycholate, pH 7.5) were mixed and allowed to incubate at room temperature (RT) for 1 h; 20 µl of ACE2-His (2.5 ng/µl) were added and incubation resumed for 1 h; wells were washed three times with immune buffer and blocked with Blocking Buffer 2 for 10 min; 100 µl of Anti-His-HRP were added to all wells which were incubated for 1 h; wells were emptied, washed three times and blocked before the addition of 100 µl of freshly prepared HRP chemiluminescent substrates; the luminescence intensity of the samples was measured in a BioTek Synergy 2 microplate reader. The luminescence values in the presence of a test compound were expressed as % of the values obtained in the absence of the compound.

### Immunoblotting

For the immunoblotting (IB) experiments, 2.5 × 10^6^ cells in 4 ml of complete medium were seeded in 60 mm plates. After a 24-h incubation, they were transfected, processed, and treated as described above under “Transfection of HEK293 cells”. Cell monolayers were washed with PBS; they were scraped off the plates and suspended in 1 ml of PBS; they were rinsed with PBS through two cycles of centrifugation at 1020 *g* for 3 min. Cell pellets were lysed in 50 µl of IP buffer [PBS, 0.3% n-dodecyl β-D-maltoside (DDM), 20 mM phenylmethylsulfonyl fluoride (PMSF), 1x Protease Inhibitor Cocktail (PIC)]; lysates were dispersed through a 30-sec on/30-sec off cycle of sonication in a water bath at 4 °C for 3 min at high intensity using a Diagenode sonicator (UCD-200TM). Protein concentrations were determined using the Bio-Rad Bradford Protein Assay kit.

For IB, cell lysates (10 µg) were supplemented with 0.25 volumes of 4× Laemmli Sample Buffer and 0.1 volume of β-mercaptoethanol (final 10% v/v); they were heated at 55 °C for 10 min. Proteins were fractionated on an 8% or 10% SDS-PAGE and electrophoretically transferred onto a polyvinylidene fluoride (PVDF) membrane. The membrane was blocked in Tris-buffered saline (TBS, 50 mM Tris-HCl, 150 mM NaCl, pH 7.6) containing 5% milk-0.1% Tween (blocking solution) for 1 h. It was first incubated overnight at 4 °C with a primary antibody in a 5% bovine serum albumin (BSA) solution and then for 1 h at room temperature, with a horseradish peroxidase (HRP)-conjugated secondary antibody in blocking solution. The membrane was probed for HRP reaction using Bio-Rad Clarity Western ECL Substrate Revelation Kit. Bands were captured using a Bio-Rad ChemiDoc Imaging System and analyzed by densitometry with the Bio-Rad Image Lab Software. The densitometric values for ACE2, Spike-GFP bands were normalized by those for β-actin taken as an internal standard of protein content.

### Furin in vitro assay

The assay was conducted using the BPS Bioscience Furin Protease Assay Kit according to the manufacturer’s protocol. Briefly, a 50-µl reaction mixture in 25 mM Tris/1 mM CaCl_2_ 0.5% w/v Brij-35, pH 9.0 (assay buffer) was prepared with 50 ng of recombinant soluble human furin, 10 µl of quercetin at different concentrations in 10% DMSO (1% final); the mixture was kept at room temperature for 30 min; it was then supplemented with 50 µl of 4 µM of the furin substrate PyroGlu-Arg-Thr-Lys-Arg-4 methylcoumaryl-7-amide (Pyr-RTKR-AMC) in the assay buffer, and further incubated for 30 min at room temperature. Fluorescence intensity resulting from substrate cleavage was measured at an excitation of 380 nm and an emission of 460 nm using a Tecan Infinite M1000 microplate reader. Quercetin inhibitory activity is expressed in % relative to blank (100%). Decanoyl-Arg-Val-Lys-Arg-chloromethylketone (Dec-RVKR-CMK), a confirmed furin inhibitor, was used as a positive control of inhibition.

### Statistical analysis

Quantitative values are expressed as means ± standard deviation (SD). They were calculated and statistically compared by one-way ANOVA using GraphPad Prism version 9.5.1. Significance of differences between experimental groups was set at *p* < 0.05.

## Results

### Quercetin inhibits SARS-CoV-2 infection of VeroE6 and Caco2 cells

**Fig. 1:**
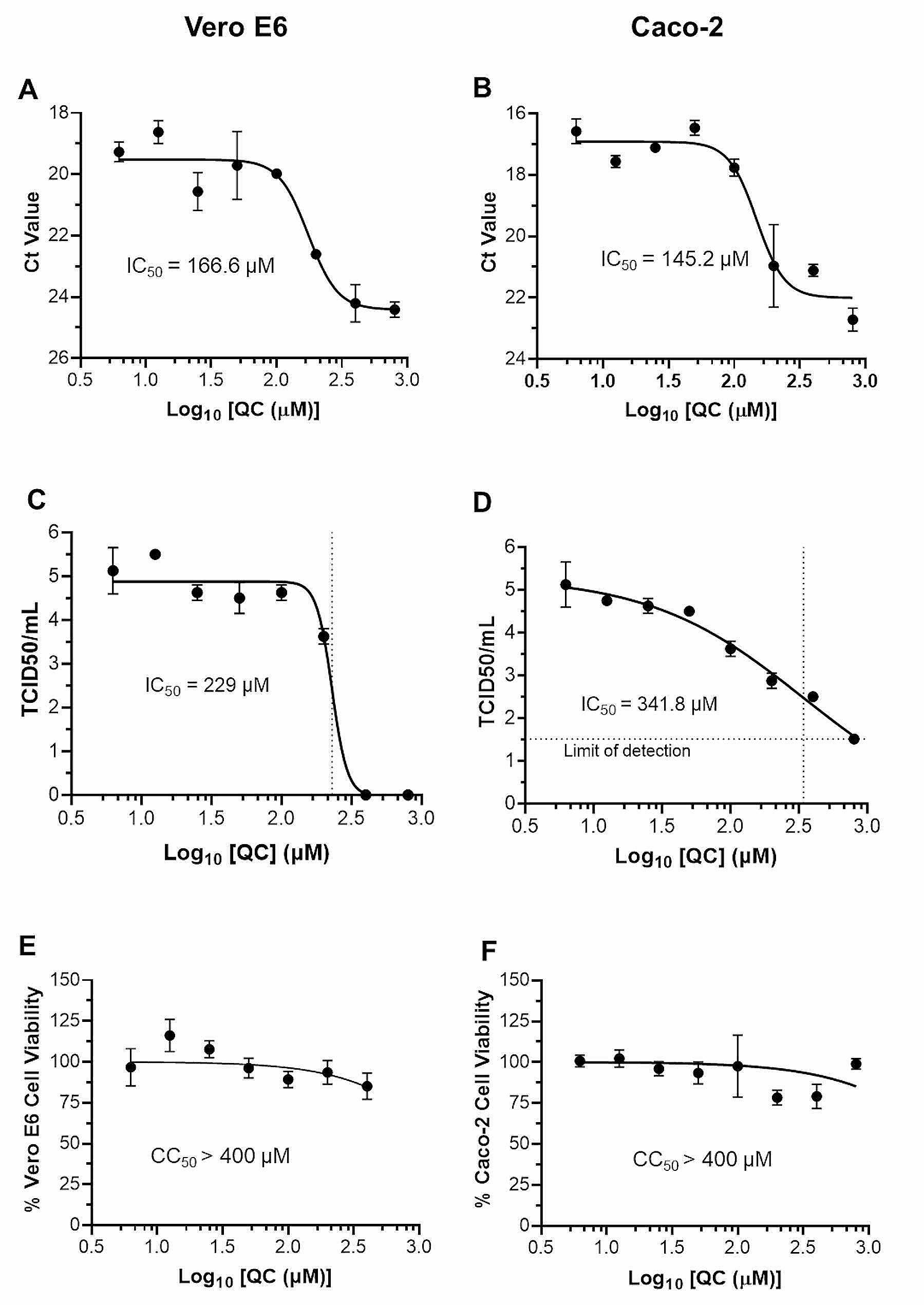
Quercetin inhibits SARS-CoV-2 infection. Vero E6 (**A**) or Caco2 (**B**) cells were pre-treated with various concentrations of quercetin (ranging from 6.25 µM to 400 µM) before being infected with SARS-CoV-2 at an MOI of 0.1. Supernatants were harvested after 48 h, and virus RNA was quantified via RT-qPCR. RNA levels are expressed on an inverse y-axis as Ct (cycle threshold) values. Results were obtained from three independent experiments and are expressed as mean Ct values ± SD (**C** and **D**). Cell viability after 48 h incubation with quercetin in the same concentration range was determined for Vero E6 (**E)** and Caco2 (**F**) cells using the CyQUANT™ XTT Cell Viability Assay kit

To determine whether quercetin could inhibit virus infection, Vero E6 and Caco-2 cells were infected with SARS-CoV-2 and treated with quercetin at different concentrations ranging from 6.25 µM to 400 µM. Two days post-infection (dpi), supernatants were collected, and viral RNA was quantified by RT-qPCR. The Ct values increased with increasing concentrations of quercetin, reflecting a decrease in viral genomes released, i.e., an inhibition of virion production. The half inhibitory concentration (IC_50_) of the inhibition in Vero E6 and Caco-2 cells was 166.6 µM (Fig. 1A) and 145.2 µM (Fig. 1B), respectively.

The titer of infectious particles in culture supernatants following quercetin treatment of infected cells was assessed by determining their TCID_50_/ml: it was 229 µM and 341.8 µM for Vero E6 (Fig. 1C) and Caco-2 (Fig. 1D) cells, respectively.

The quercetin concentrations tested did not induce any significant loss of viability of either cell type, giving a half cytotoxic concentration (CC_50_) > 400 µM (Fig. 1, E and F), and a Selectivity Index (SI, CC_50_/IC_50_) of > 2.4.

### Quercetin inhibits the formation of syncytia by S and ACE2 co-expressing cells

Since some of quercetin bioactivities has been ascribed to its disturbance of the cellular membrane lipid bilayer by intercalating into it [26, 27], we surmised that this intercalation might impair not only the interaction between the viral S protein and cellular ACE2, but also the topology and activity of membrane receptors and enzymes which facilitate membrane fusion and viral entry. We therefore set up a HEK293 cell-based assay (without infectious virus) to examine the effect of quercetin on syncytium formation that results from S protein-ACE2 interaction.

**Fig. 2:**
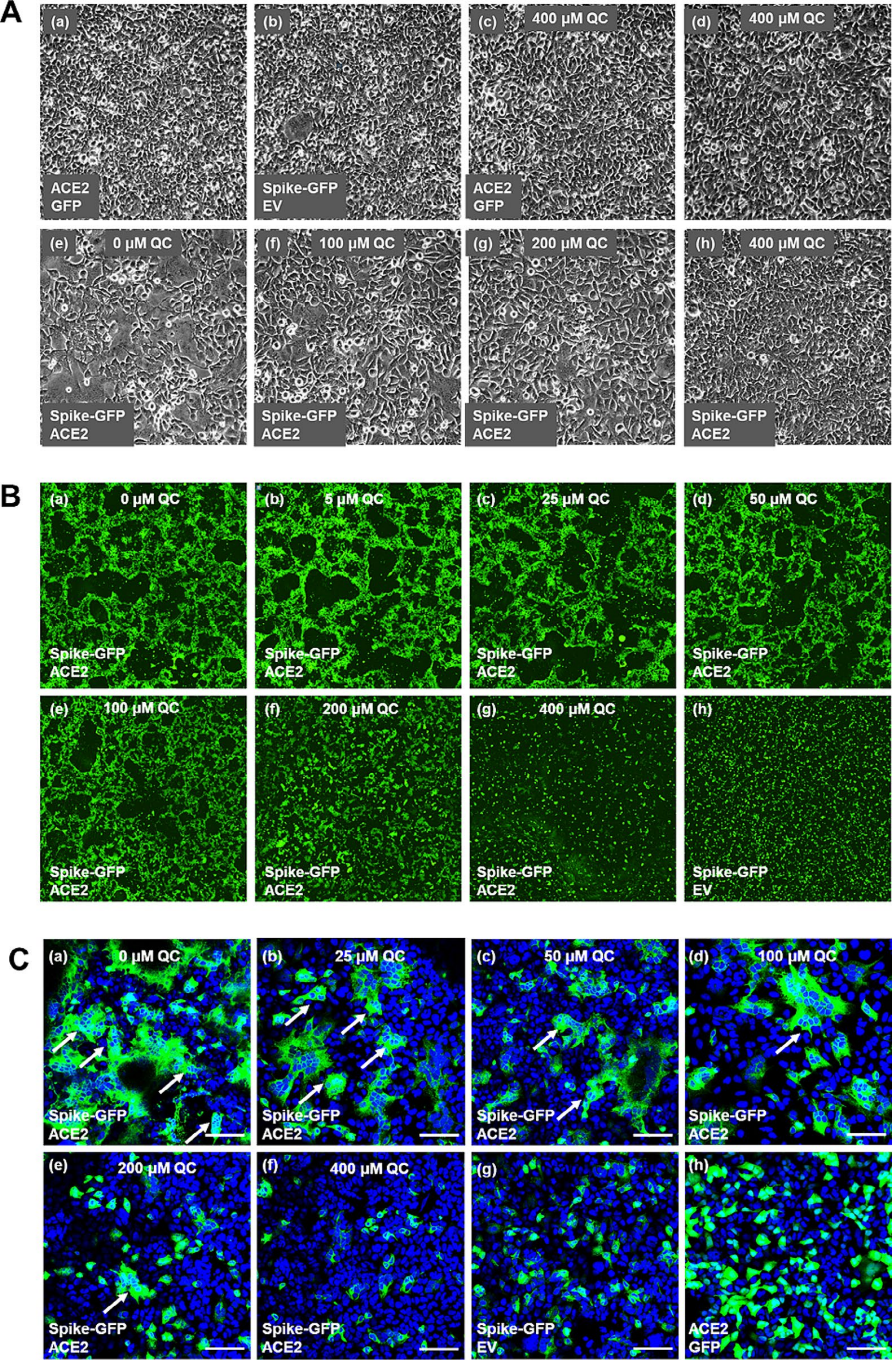
Microscopy of HEK293(S + ACE2) cell syncytialization and its inhibition by quercetin. (**A**) Brightfield microscopy. Controls cells (a-d) were transfected with plasmid vector for expression of indicated proteins and later cultured for 20 h in medium containing either DMSO (a, b) or 400 µM quercetin in DMSO. Experimental HEK293(S + ACE2) cells (e-h) were cultured for 20 h in medium containing either DMSO (e) or the indicated concentrations of quercetin (f-h). (**B**) Fluorescence microscopy. Control and experimental cells were treated as in A; GFP fluorescence was camera-captured in the Incucyte. (**C**) Confocal microscopy. Controls and experimental cells were treated as A, fixed, and stained with Hoechst. Images were captured. Arrows point to clusters of nuclei within region intense GFP fluorescence. Scale bar: 100 μm

When HEK293 cells were transfected with the pS-GFP or the pACE2 plasmid vector, cell monolayers preserved their normal spread-out morphology as visible by brightfield microscopy (Fig. 2A, a, b). Addition of 400 µM to pACE2-transfected cells or untransfected did not alter this morphology (Fig. 2A, c, d). When, on the other hand, cells were transfected with both pS-GFP and the pACE2 vectors [heretofore named HEK293(S + ACE2) cells], the monolayer was characterized by groupings of cells around denuded areas, suggesting the formation of syncytia (Fig. 2A, e). Addition of quercetin (100 to 400 µM) gradually restored the normal morphology of monolayers (Fig. 2A f-h). This was confirmed by examining the distribution of GFP fluorescence (Fig. 2B). When S-GFP was expressed alone, punctate fluorescent cells were observed around the plate (Fig. 2B, h). With HEK293(S + ACE2) cells, intense fluorescence surrounding dark areas were observed (Fig. 2B, a). In the presence of increasing concentrations of quercetin, the fluorescence intensity gradually decreased and became punctate again (Fig. 2B, b-g).

The syncytialization of HEK293(S + ACE2) cells was ascertained by confocal microscopy. As shown in Fig. 2C, a, areas of intense GFP fluorescence were characterized by clusters of blue-stained nuclei, as can be expected of syncytia (arrows). The clusters gradually decreased in number and fluorescence intensity with the addition of increasing concentrations of quercetin (Fig. 2C, b-f). Such clusters were not observed when cells were pS-GFP or pACE2 alone (Fig. 2C, g-h)

### Quercetin IC_50_ of syncytialization of HEK293(S + ACE2) cells, CC_50_ of HEK293 cells, and IC_50_ of in vitro binding of ACE2 to S-RBD

**Fig. 3:**
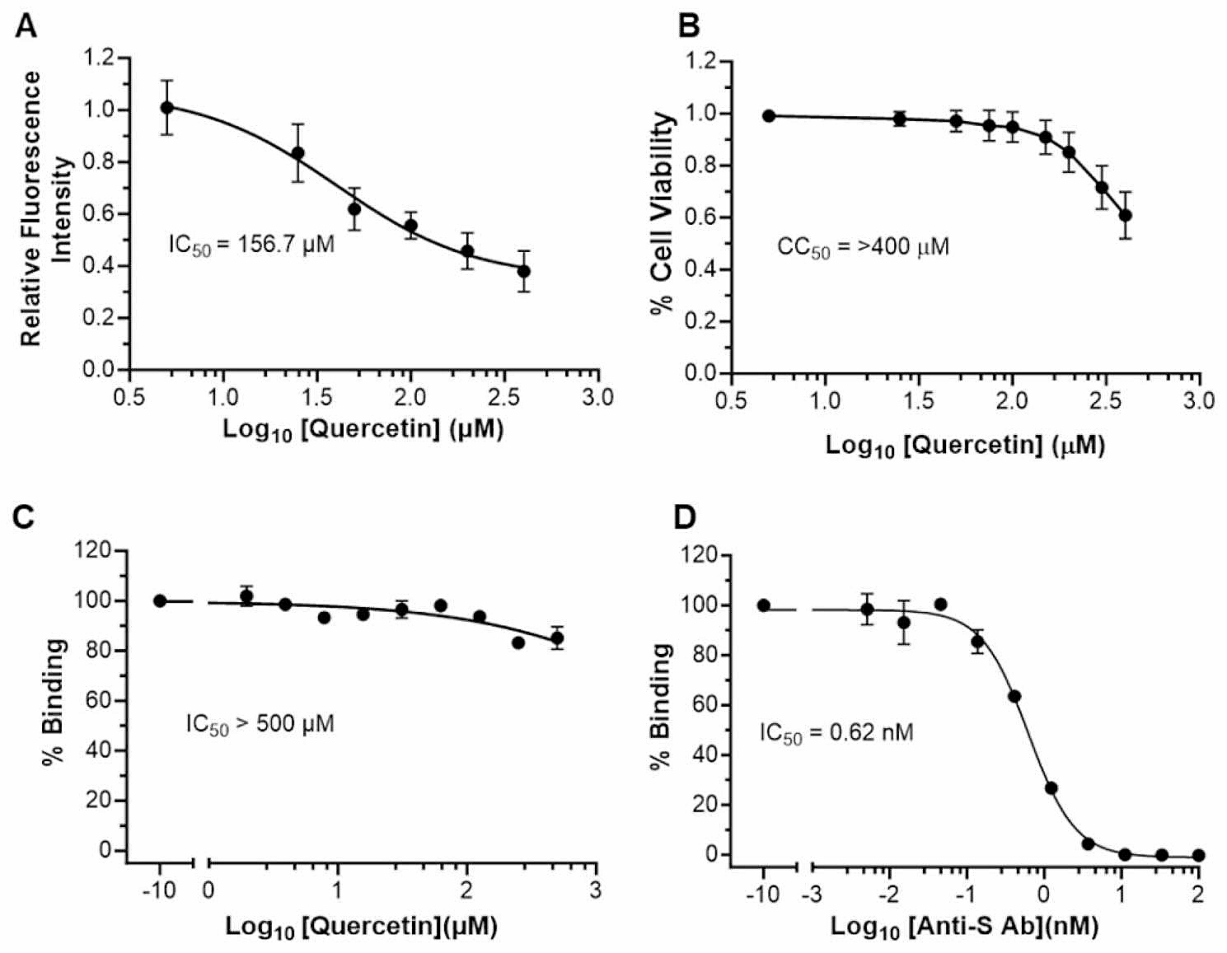
Quercetin IC_50_ and CC_50._ (**A**) IC_50_ of quercetin inhibition of syncytialization of HEK293(S + ACE2) based on decrease of GFP fluorescence. (**B**) CC_50_ of quercetin on HEK293 cells as determined using the MTS viability assay. (**C**) IC_50_ of quercetin inhibition of in vitro binding of ACE2 to Spike RBD. (**D**). IC_50_ of anti-Spike antibody inhibition of in vitro binding of ACE2 to Spike RBD. Results are from 3 separate experiments and are expressed as means ± SD

Since GFP fluorescence intensity correlated with the abundance of syncytia, its integrated intensity per image was measured and used to derive a IC_50_ of quercetin: it was found to be 156.7 µM (Fig. 3A). In a separate experiment, MTS viability assay was applied to HEK293 cells in the same concentration range to derive a CC_50_ of quercetin: it was above the highest concentration tested (> 400 µM, Fig. 3B).

Syncytialisation inhibition could be due to quercetin interference in the binding of the viral Spike protein to cellular ACE2 as inferred from *in silico* docking studies [15, 16]. We examined this possibility in an in-vitro assay of binding of Spike protein receptor-binding domain (RBD) to recombinant ACE2. Quercetin up to 300 mM failed to inhibit this binding (Fig. 3C) whereas an anti-S antibody very efficiently did so (Fig. 3D).

### Quercetin inhibits the production of the S2’ fragment of the spike protein

**Fig. 4:**
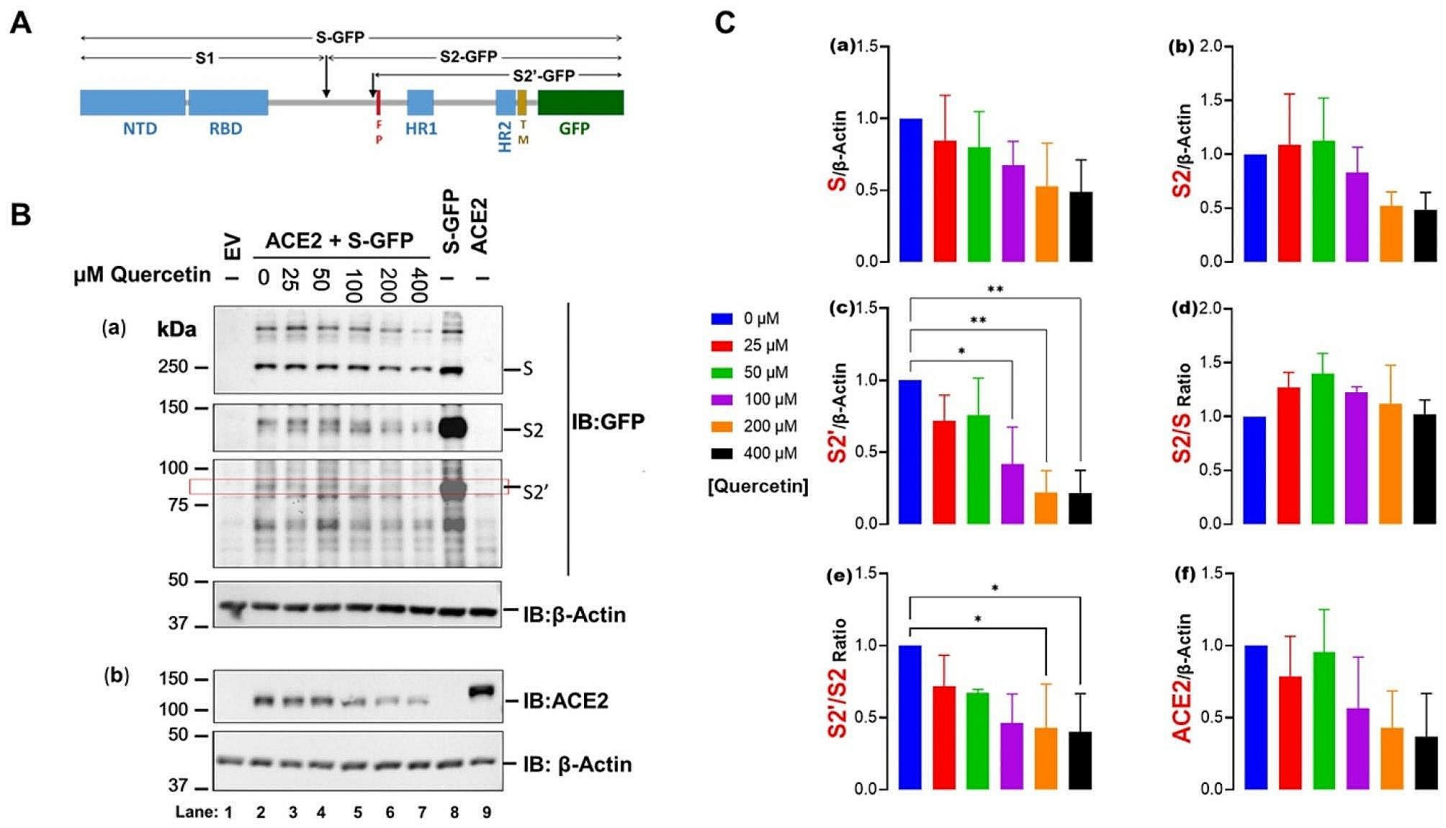
Immunoblotting of ACE2 as well as S and S-related proteins in HEK293(S + ACE2) cells treated with quercetin. (**A**) Diagrammatic representation of the S-GFP fusion protein and its proteolytic fragment. Acronyms: NTD, N-terminal domain; RBD, receptor binding domain; FP, fusion peptide; HR, heptad region, TM, transmembrane domain, GFP, green fluorescent protein. (**B**) Representative Immunoblot of cells extracts transfected with empty vector (EV) (lane 1), vector for expression of S-GFP (lane 8), ACE2 (lane 9), or both S-GFP and ACE2 (lanes 2–7) with DMSO (lane 2) or increasing concentrations of quercetin (lanes 3–7); (a) The blot was immuno-probed successively for GFP and β-actin; (b) a parallel blot of the same cell extracts was immune-probed successively for ACE2 and β-actin antibody. (**C**) Densitometry of immunoreactive bands in three separate experiments was conducted. S, S2 and S2’and ACE2 values were normalized by those of β-actin (a-c, f). The ratios S2/S (d) and S2’/S2 (e) were computed as measures of the proteolytic conversion S to S2 and S2 to S2’, respectively. Values are expressed as means ± SD. * < 0.05, ** < 0.005

The S-GFP expressed is the transmembrane spike protein C-terminally fused to GFP (Fig. 4A). In immunoblotting of extracts of HEK293 cells expressing the fusion protein, an anti-GFP antibody should recognize the S protein as well as membrane-bound N-terminally cleaved products, most prominently membrane-bound S2 and S2’. This was indeed the case, as shown in Fig. 4B, (lane 8). The identity of the immune bands was confirmed via immunoblotting cell lysates from cells transfected with a vector for producing unfused S protein or S-GFP fusion protein, using two different polyclonal antibodies raised against the native S (Supplementary Fig. S1).

In HEK293(S + ACE2) cells, expression of S and its processed fragments was consistently less robust compared to cells expressing S-GFP alone (complemented with an empty vector to equalize the total amount of vector DNA; Fig. 4B, a, lane 2 vs. lane 8). The addition of quercetin to the doubly transfected cells reduced the expression of all these S forms in a concentration-dependent manner (Fig. 4B, a, lanes 2–7).

ACE2 expression in HEK293(S+ ACE2) cells was similarly affected by the double transfection (Fig. 4B, b, lane 2 vs. lane 9) and quercetin addition (Fig. 4B, b, lanes 2–7). Shed ACE2 in spent media was titrated by ELISA: the decrease in media paralleled that in cells (not shown), suggesting that quercetin did not affect shedding in any way.

The optical density of immunoreactive bands corresponding to S, S2, S2’, ACE2 was measured and normalized relative to that of β-actin (Fig. 4C). The quercetin-induced decrease in S and S2 expression did not reach significance (Fig. 4C, a-b); however, the decrease in S2’expression at 100 µM quercetin and above was significant (Fig. 4C, c).

S2 and S2’ are generated through cleavage by furin after specific basic motifs [28]. S2/S and S2’/S2 ratio were computed from their normalized values to assess the effect of quercetin on these two major proteolytic events. A significant decrease in the conversion of S2 to S2’ was noted at 200 and 400 µM (Fig. 4C, e). This conversion exposes an N-terminal hydrophobic motif called fusion peptide (FP, in Fig. 4A) which facilitates fusion among cells.

Extracts of HEK293(S + ACE2) cells were subjected to immunoprecipitation with an anti-GFP antibody and the precipitates subjected to immunoblotting using either the anti-GFP antibody or an anti-ACE2 antibody. The results showed that addition of quercetin did not significantly change the ratio of S or S2 to ACE2 pulled down by the antibody (Supplemental Fig. S2), suggesting that the flavonol did not interfere with binding between the two molecules, in concordance with the results of the in vitro binding assay using recombinant S-RBD and ACE2 (see Fig. 3C).

### Quercetin inhibits furin in vitro

**Fig. 5:**
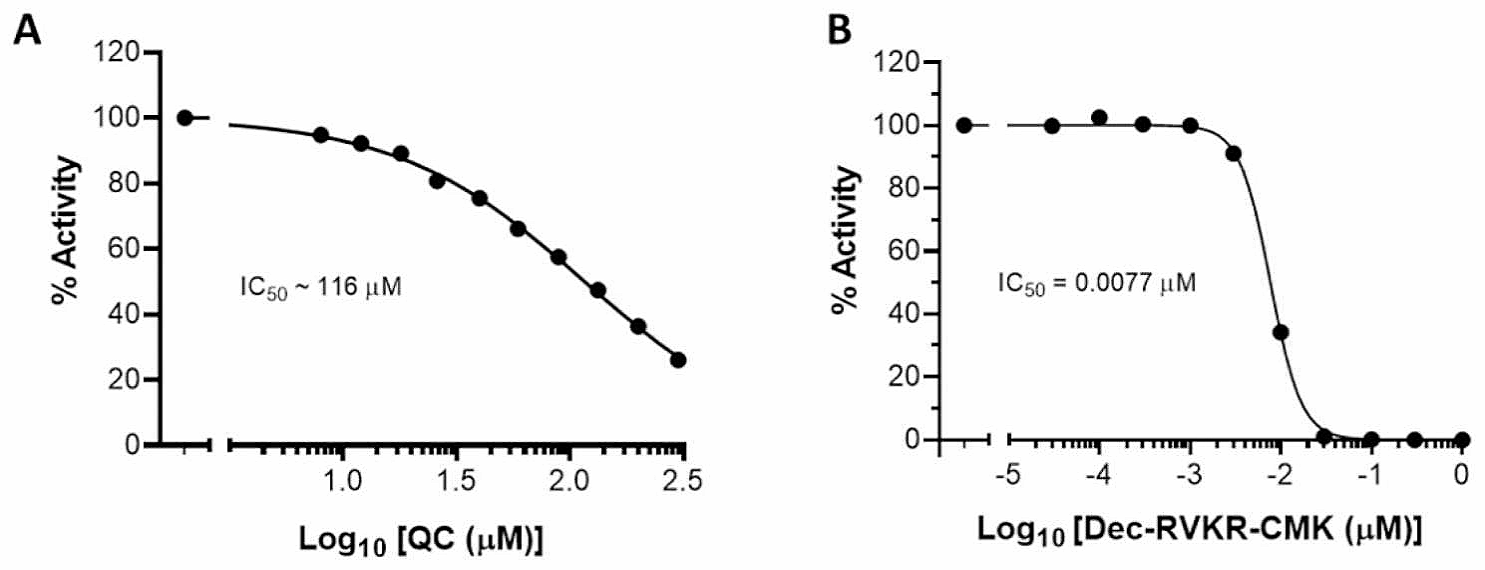
Quercetin inhibition of furin activity. As described under Materials and Methods, furin proteolysis of the fluorogenic Pyr-RTKR-AMC substrate was assayed in absence or the presence of increasing concentrations of: (**A**) quercetin or (**B**) the synthetic Dec-RVKR-CMK inhibitor

Zhu and coll. have examined the inhibitory effect of several polyphenols, including quercetin, on furin proteolysis of its fluorogenic substate Pyr-RTKR-AMC, in the concentration range of 0 to 40 µM: they found that quercetin inhibited this activity by about 45% at 40 µM [18]. Using a similar in vitro furin assay and extending the concentration range (0 to 300 µM), we determined the IC_50_ of inhibition to be about 116 µM (Fig. 5A). The IC_50_ of Dec-RVKR-CMK canonical furin inhibitor was 7.7 nM under the same conditions (Fig. 5B), suggesting that inhibition by quercetin may be through mechanism not involving interaction with the furin catalytic site.

## Discussion

This study demonstrates that quercetin, at concentrations above 100 µM can inhibit SARS-CoV-2 infection of Vero E6 and Caco-2 cells. The term ‘infection’ refers broadly to any step of the virus life cycle — including attachment, entry, genome replication, gene transcription, assembly, and egress — all of which are necessary for the production of infectious progeny virions. Quantification of the levels of virus genomic RNA was used as a surrogate measure of virus replication. Their reduction in the presence quercetin suggests that cell exposure to this flavonol negatively impacts virus replication.

These effective concentrations of quercetin in this study are 2-order of magnitude above those ordinarily found in blood following oral administration of quercetin to animals or humans [29], putting into question the ability of this flavonol to block infection by the virus in vivo. However, a few studies have shown that lungs, the tissues primarily infected by SARS-CoV-2, were among the organs where quercetin and its metabolites mostly accumulated after such an administration [30, 31]. Moreover, experimental evaluation of antiviral activity of green tea extracts in mice and humans have provided evidence that concentrations of epigallocatechin 3 − 0 gallate (EGCG), the active phytochemical and a polyphenol like quercetin, could reach and surpass 100 µM in saliva or pharyngeal swabs, 3 min to 2 h after oral administration [32–34], raising the possibility that the upper respiratory tract could similarly accumulate quercetin with the choice of a proper mode of administration (e.g. oral or nasal spray).

In this study, the IC_50_ of quercetin inhibition of SARS-CoV-2 replication in Vero E6 and Caco2 cells was about 150 µM as determined by RT-qPCR of viral RNA released in the medium. In contrast, Kandeil et al. [35] reported that the flavonol inhibited SARS-CoV-2 infection of Vero E6 with an IC_50_ of 18.2 µM, as assessed by a plaque reduction assay. Using human lung epithelial Calu-3 for infection and a similar Vero E6 cell-based plaque-forming assay, Chaves et al. [36] determined the IC_50_ of quercetin inhibition to be 2.4 µM. This discordance in IC_50_ results could be explained by differences of viral strain, cell line, and MOI used for infection, among other factors.

The binding of SARS-CoV-2 Spike protein to human ACE2 is the prelude to viral entry into and infection of human cells. When the S protein is newly biosynthesized within the infected cells, it is translocated to the plasma membrane where it interacts with the ACE2 of adjacent cells, initiating the formation of syncytia [37]. Both S and ACE2 are type-1 transmembrane proteins; both can be susceptible to proteolytic cleavages by membrane proteases, releasing the N-terminal fragment into the extracellular space. Furin and TMPRSS2 mediate this endoproteolysis: furin is the primary enzyme that converts S to N-terminal S1 and transmembrane S2 proteins, and of the latter to transmembrane S2’ protein; whereas TMPRSS2 mediates the shedding of the ACE2 ectodomain following its binding to the S protein [28]. The N-terminal hydrophobic FP of S2’ promotes membrane fusion and syncytium formation [38]. The orchestration of these proteolytic events may explain the apparent lower level of both S and ACE2 when they are co-expressed in HEK293 cells compared to when either protein is expressed alone. A decrease in endogenous ACE2 expression following transfection of SARS-CoV-2 spike protein has also been noted in Vero E6 cells [39].

Addition of quercetin at 100 to 400 mM interferes with syncytium formation by HEK293(S + ACE2) cells. This inhibition occurs with an IC_50_ of 156.7µM. In contrast, using HEK293-IIIA cells expressing S and ACE2, Singh et al. [39] reported that quercetin at 10 µM significantly reduced the size (by ~ 80%) and area (by ~ 60%) of syncytia, as determined by brightfield image analysis. Why syncytialization inhibition by the flavonol appears to be greater in this cell line compared to the one used in this study is unclear. Like its effect on apoptosis [40], the IC_50_ of quercetin inhibition of syncytium formation can significantly vary among cell lines.

Quercetin addition to HEK293(S + ACE2) cells had little effect on the conversion of S to S2, but it significantly reduced the conversion of S2 to S2’, suggesting it inhibited the enzyme(s) responsible. Furin is one the enzymes mediating this conversion [28]. We found that quercetin inhibits furin cleavage of a specific synthetic substrate in vitro, in a concentration-dependent manner and with an IC_50_ of 116 µM. This IC_50_ value is not far removed from that obtained in HEK293(S + ACE2) syncytialization assay (156.7 µM) as well as those obtained in the SARS-CoV-2 infectivity assay with Vero E6 (166.6 µM) or Caco2 (145.2 µM) cells. In this context, it is worth mentioning that in vitro inhibition of furin by polyphenols has been attributed, not to inactivation of its catalytic site, but to the formation of quinone oxidation products that interact non-specifically with the furin protein, changing its conformation and reducing its enzymatic activity [41].

It is also possible that the intercalation of quercetin into the lipid bilayer perturbs the topology of membrane enzymes [42], preventing the binding of S2 protein to the catalytic pocket of furin. Interestingly, quercetin-3-glucoside—also known as isoquercetin or isoquercitrin—which is less hydrophobic than quercetin, and thus less membrane-intercalating, failed to inhibit syncytium formation (Supplemental Fig. S3).

## Conclusion

Data presented in this report indicate that quercetin can inhibit SARS-CoV-2 replication in Vero E6 and Caco-2 cells. Results from experiments with surrogate HEK293(S + ACE2) cells suggest that, mechanistically, it prevents the syncytium formation which normally follows initial expression of viral proteins in infected cells, facilitating virion propagation. At the molecular level, quercetin reduction of furin-mediated production the S2’ fusogenic fragment might contribute to the diminished syncytialization. The inhibition of syncytium formation and of S2’ fragment production observed in the surrogate HEK293 (S + ACE2) cells remains to be demonstrated in virus-infected cells. Nonetheless, the present data support the possibility that quercetin could be an anti-COVID-19 drug. Its in vivo efficacy should be evaluated experimentally on appropriate animal models and, ultimately, in clinical trials on human patients.

### Electronic supplementary material

Below is the link to the electronic supplementary material.

**Supplementary Material 1:** Supplementary Figure S1. Confirmation of S protein bands. Cells were transfected with the indicated expression vectors and their extracts analyzed as described for Fig. 3. Immunoblotting of S protein and its fragments was performed using antibodies from Abcam (cat# ab272504) and Sino Biological (cat# 40592-T62). The Spike-Linker-GFP gene is expressed as a fusion S-GFP protein whereas with the Spike-P2A-GFP gene, the S protein and GFP are expressed as two separate molecules, hence the size difference in immunoreactive S bands produced par the two vectors.

**Supplementary Material 2:** Supplementary Figure S2. Pull-down of ACE2 by S protein. HEK293(S+ACE2) cell extracts were subjected to immunoprecipitation with GFP-trap beads. The precipitates were analyzed by immunoblotting for ACE-2 and GFP; the densities of immunoreactive bands were determined. A. A representative blot. B&C. The S/ACE2 and S2/ACE density ratios were computed. The values (means ± SD of 3 independent experiments) of quercetin-treated cells were expressed relative to those of DMSO treated control cells.

**Supplementary Material 3:** Supplementary Figure S3. Effect of isoquercetin on HEK293(S+ACE2) syncytialization. The experiment was conducted as described in Fig. 1. Isoquercetin did not inhibit the formation de syncytia.

**Supplementary Material 4:** Supplementary Table S1. List of reagents, sources and catalog numbers

**Supplementary Material 5:** Supplementary Materials and Methods

## Data Availability

No datasets were generated or analysed during the current study.

## Abbreviations

3CL^pro^: 3-Chymotrypsin-like protease
ACE2: Angiotensin-converting enzyme 2
ANOVA: Analysis of variance
BSA: Bovine fetal albumin
CatB: Cathepsin B
CatL: Cathepsin L
COVID-19: Coronavirus disease of 2019
DDM: n-Dodecyl-β-D-maltoside
DMEM: Dulbecco’s Modified Eagle Medium
DMSO: Dimethylsulfoxide
DPI: Days post-infection
eGFP: Enhanced GFP
EV: Empty vector
FBS: Fetal bovine serum
FP: Fusion peptide
GFP: Green fluorescent protein
HEK: Human embryonic kidney
HRP: Horseradish peroxidase
IB: Immunoblotting
IP: Immunoprecipitation
MEM: Minimal Essential Medium
MOI: Multiplicity of infection
M^pro^: Main protease
NML: National Microbiology Laboratory
OD: Optical density
PBS: Phosphate-buffered saline
PHAC: Public Health Agency of Canada
PIC: Protease inhibitor cocktail
PL^pro^: Papain-like protease
PMSF: Phenylmethylsulfonyl fluoride
PVDFv: Polyvinylidene difluoride
QC: Quercetin
RBD: Receptor binding domain
RdRp: RNA-dependent RNA polymerase
RT-qPCR: Reverse transcription-polymerase chain reaction
SARS-CoV-2: Severe acute respiratory syndrome-coronavirus 2
SD: Standard deviation
TBS: Tris-buffered saline
TMPRSS2: transmembrane protease serine 2
