# Supplementary figures and images for: Quercetin inhibits SARS-CoV-2 infection and prevents syncytium formation by cells co-expressing the viral spike protein and human ACE2
Source: Virol J. 2024 Jan 25;21:29. doi: 10.1186/s12985-024-02299-w (PMC10811921; doi:10.1186/s12985-024-02299-w)

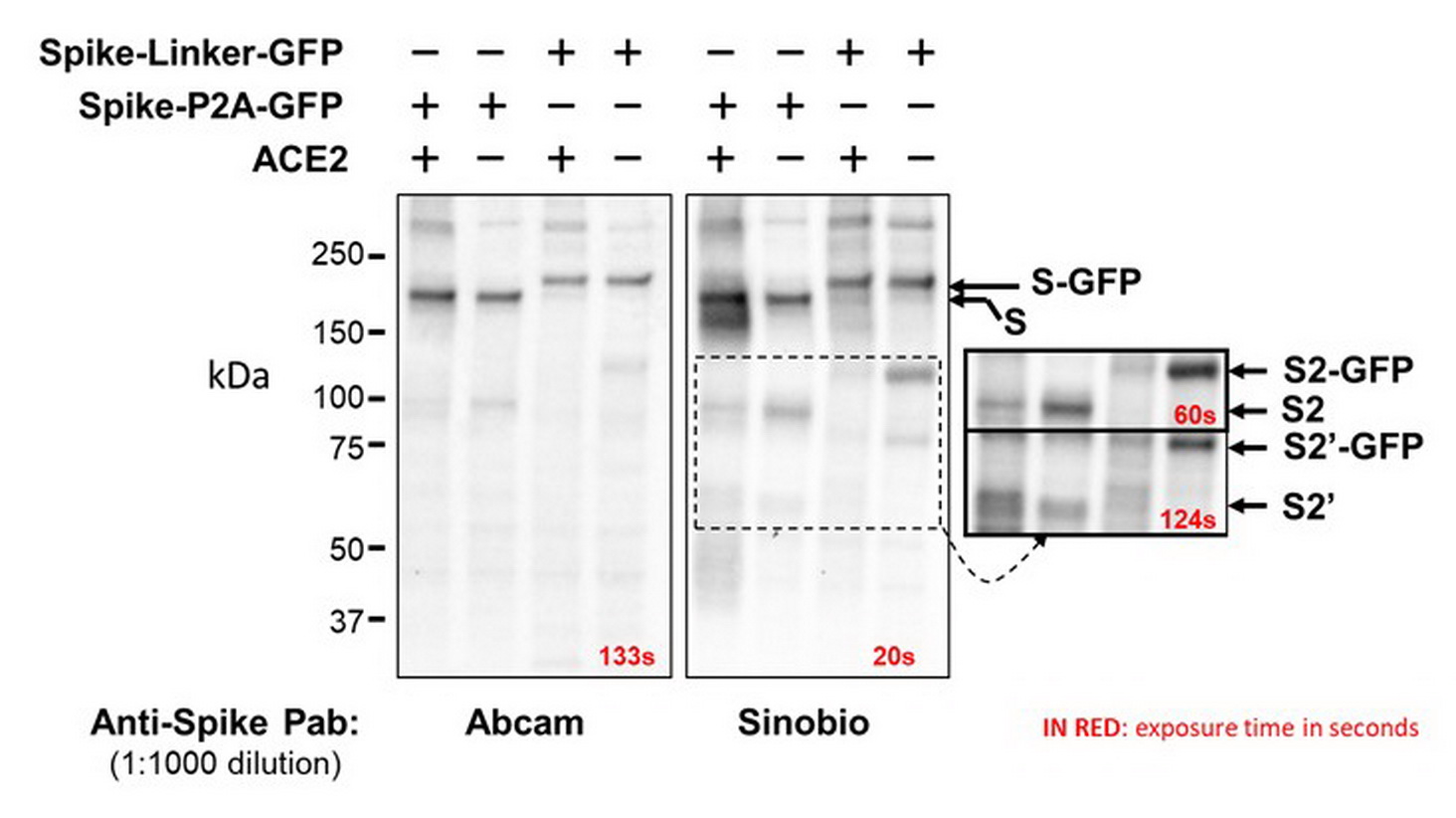

Supplement: Supplementary file 1 — Supplementary Material 1: Supplementary Figure S1. Confirmation of S protein bands. Cells were transfected with the indicated expression vectors and their extracts analyzed as described for Fig. 3. Immunoblotting of S protein and its fragments was performed using antibodies from Abcam (cat# ab272504) and Sino Biological (cat# 40592-T62). The Spike-Linker-GFP gene is expressed as a fusion S-GFP protein whereas with the Spike-P2A-GFP gene, the S protein and GFP are expressed as two separate molecules, hence the size difference in immunoreactive S bands produced par the two vectors. [file 12985_2024_2299_MOESM1_ESM.jpg]

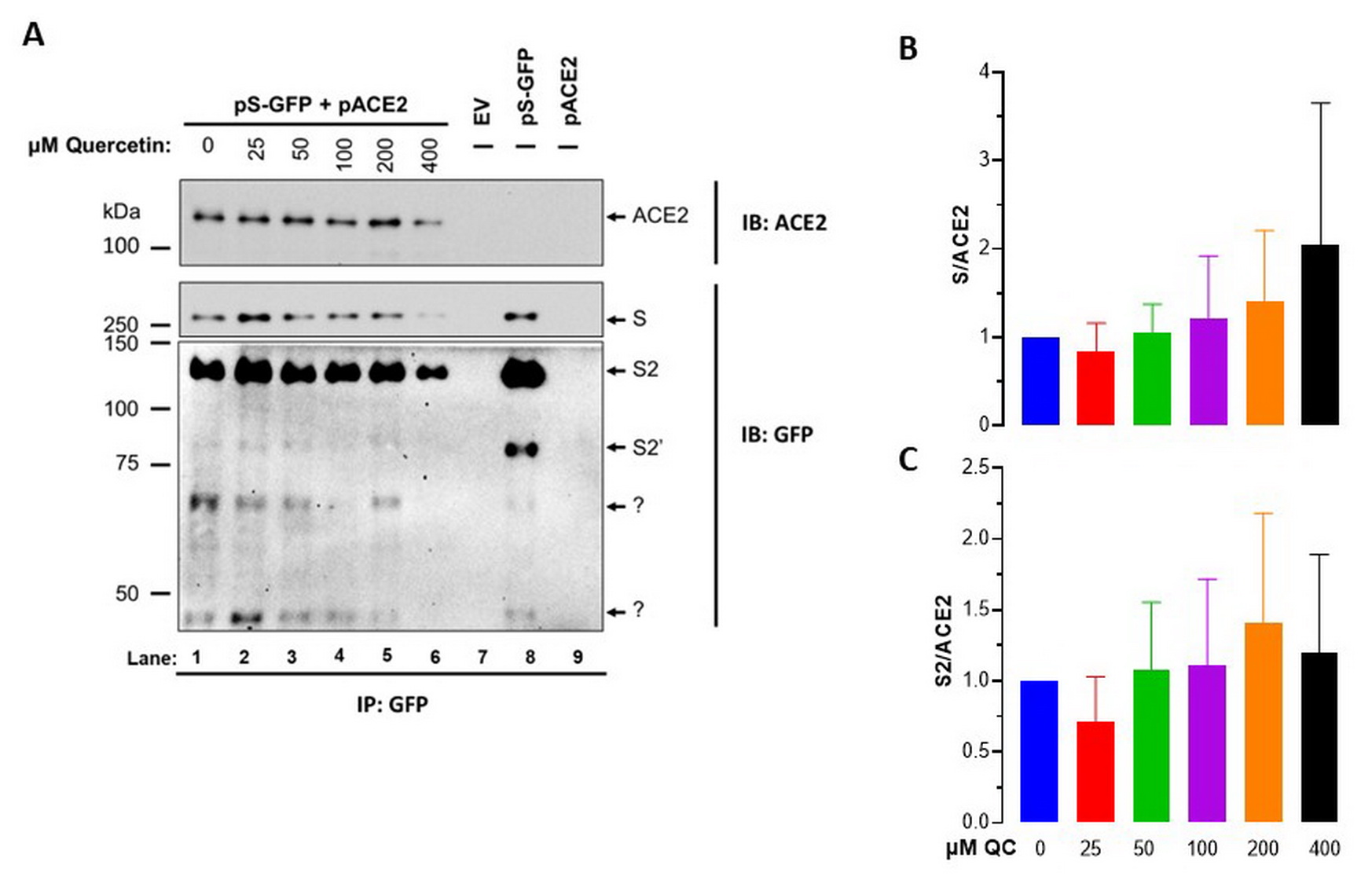

Supplement: Supplementary file 2 — Supplementary Material 2: Supplementary Figure S2. Pull-down of ACE2 by S protein. HEK293(S+ACE2) cell extracts were subjected to immunoprecipitation with GFP-trap beads. The precipitates were analyzed by immunoblotting for ACE-2 and GFP; the densities of immunoreactive bands were determined. A. A representative blot. B&C. The S/ACE2 and S2/ACE density ratios were computed. The values (means ± SD of 3 independent experiments) of quercetin-treated cells were expressed relative to those of DMSO treated control cells. [file 12985_2024_2299_MOESM2_ESM.jpg]

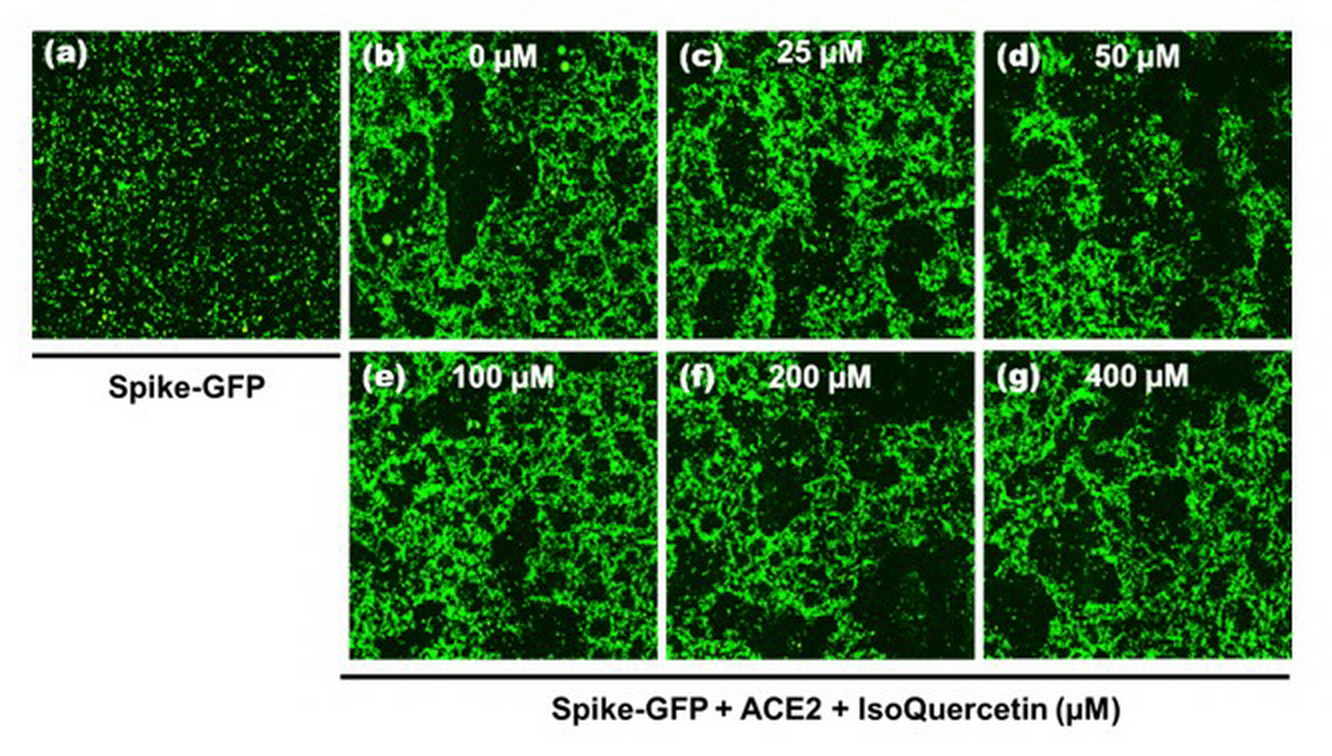

Supplement: Supplementary file 3 — Supplementary Material 3: Supplementary Figure S3. Effect of isoquercetin on HEK293(S+ACE2) syncytialization. The experiment was conducted as described in Fig. 1. Isoquercetin did not inhibit the formation de syncytia. [file 12985_2024_2299_MOESM3_ESM.jpg]
