# Supplementary material for: Quercetin inhibits SARS-CoV-2 infection and prevents syncytium formation by cells co-expressing the viral spike protein and human ACE2
Source: Virol J. 2024 Jan 25;21:29. doi: 10.1186/s12985-024-02299-w (PMC10811921; doi:10.1186/s12985-024-02299-w)
Supplement: Supplementary file 4 — Supplementary Material 4: Supplementary Table S1. List of reagents, sources and catalog numbers [file 12985_2024_2299_MOESM4_ESM.docx]

**Supplementary Table S1. List of reagents, sources and catalog numbers**

| **Reagents** | **Source (cat #)** |
| --- | --- |
| **Flavonols** |  |
| Quercetin | Sigma-Aldrich (Q4951) |
| Isoquercetin | Sigma-Aldrich (16654) |
| **Cell culture media** |  |
| Dulbecco’s modified Eagle medium (DMEM) | Gibco (11965-092) or Wisent (319-005- CL |
| Minimum essential medium (MEM) | Gibco (11095-080) |
| Heat-inactivated fetal bovine serum (hiFBS) | Gibco 25030-081) or Wisent (098450) |
| Penicillin/streptomycin (PeniStrep) | Gibco (15140-122 or Wisent (450-201-E) |
| **Plasmids** |  |
| pcDNA-3 | Invitrogen (V7920) |
| pEGFP-C1 | Clontech (6084-1) |
| pcDNA3.1-hACE2-C9 | Addgene (145033) |
| 2019-nCov-Linker_pcDNA3.1(+)-C-eGFP | MolecularCloud (MC_0101089) |
| 2019-nCov_pcDNA3.1(+)-P2A-eGFP | MolecularCloud (MC_0101087) |
| **Antibodies (used dilution)** |  |
| Goat anti-ACE2 antibody (1:200) | R&D System (AF933) |
| Mouse anti-GFP (B-2) (dil. 1:7000)) | Santacruz (sc-9996) |
| Mouse anti-actin (1:400) | Thermo Fisher (A2066) |
| HRP-conjugated rabbit anti-goat Ig (1:5000) | Sigma-Aldrich (A5420) |
| HRP-conjugated goat anti-mouse Ig (1:10000) | Thermo Fisher (31430) |
| **Kits** |  |
| jetPRIME^®^ Transfection Reagent | Polyplus (101000015) |
| CyQUANT™ XTT Cell Viability Assay Kt | Thermo Fisher (X12223) |
| MTS Cell Proliferation Assay Kit | Abcam (ab197010) |
| QIAamp Viral RNA Mini Kit | Qiagen (52906) |
| LightCycler RNA Master Hydrolysis Probes kit | Roche (04991885001) |
| Bradford Protein Assay Kit | Bio-Rad (5000006) |
| Clarity Western ECL Substrate Revelation Kit | Bio-Rad, (1705061) |
| Blocking Buffer 2 | BPS Bioscience (79728) |
| Furin Protease Assay Kit | BPS Bioscience (78040) |
| **Others** |  |
| Recombinant soluble human furin | R&D (1503-SE) |
| Furin substrate (Pyr-RTKR-AMC), | R&D (ES013) |
| Furin inhibitor (Dec-RVKR-CMK) | BPS Bioscience (78713) |
| Dimethylsulfoxide (DMSO) | Sigma-Aldrich (D2650) |
| n-Dodecyl β-D-maltoside (DDM) | Sigma-Aldrich (D4641) |
| Phenylmethylsulfonyl fluoride (PMSF) | BDH (44217) |
| Protease inhibitor cocktail (PIC) | Roche (04693124001) |
| ChromoTek GFP-Trap® Agarose beads | Proteintech (gta-20) |
| Hoechst 33343 | Thermo Fisher (H3570) |
