# Supplementary material for: Quercetin inhibits SARS-CoV-2 infection and prevents syncytium formation by cells co-expressing the viral spike protein and human ACE2
Source: Virol J. 2024 Jan 25;21:29. doi: 10.1186/s12985-024-02299-w (PMC10811921; doi:10.1186/s12985-024-02299-w)
Supplement: Supplementary file 5 — Supplementary Material 5: Supplementary Materials and Methods [file 12985_2024_2299_MOESM5_ESM.docx]

**Supplementary material and methods**

**Immunoprecipitation**

For each 10 cell extract samples, 150 µl of GFP-Trap®-Agarose beads were conditioned by spinning at 820 *g* for 2 min and washing them twice with PBS, and then suspended in 300 µl of IP buffer (/sample). Immunoprecipitation was conducted by diluting each sample (40 µg of proteins) in 500 µl of IP buffer; adding 40 µl of GFP-Trap® agarose beads, mixing the suspension on a rotator for 1 h at room temperature, spinning down the beads at 820 *g* for 2 min, washing them twice with 500 µl of IP buffer, and thrice with cold PBS. Bead pellets were suspended in 45 µl of 1.5x Laemmli Sample Buffer containing 10% β-mercaptoethanol; they were heated at 55°C for 10 min; Aliquots (10 µl) were analyzed by immunoblotting as described in Section 2.6.

**Legends to supplementary figures**

**Figure S1. Confirmation of S protein bands.** Cells were transfected with the indicated expression vectors and their extracts analyzed as described for Fig. 3. Immunoblotting of S protein and its fragments was performed using antibodies from Abcam (cat# ab272504) and Sino Biological (cat# 40592-T62). The Spike-Linker-GFP gene is expressed as a fusion S-GFP protein whereas with the Spike-P2A-GFP gene, the S protein and GFP are expressed as two separate molecules, hence the size difference in immunoreactive S bands produced par the two vectors.

**Figure S2. Pull-down of ACE2 by S protein.** HEK293(S+ACE2) cell extracts were subjected to immunoprecipitation with GFP-trap beads. The precipitates were analyzed by immunoblotting for ACE-2 and GFP; the densities of immunoreactive bands were determined. **A.** A representative blot. **B&C.** The S/ACE2 and S2/ACE density ratios were computed. The values (means ± SD of 3 independent experiments) of quercetin-treated cells were expressed relative to those of DMSO treated control cells.

**Figure S3. Effect of isoquercetin on HEK293(S+ACE2) syncytialization.** The experiment was conducted as described in Fig. 1. Isoquercetin did not inhibit the formation de syncytia.
